# An Important New Departure

**Published:** 1885-09

**Authors:** 


### AN IMPORTANT NEW DEPARTURE.

To our apprehension there has been nothing done in the American Dental Association for years that promised so much of professional good as the appropriation of money for original research in the sections. In this the Association is doing something for scientific investigation, and is acting the part of a scientific body. No society can lay claim to being truly scientific that does not do something to encourage original study. At the meeting in Cincinnati, a prize was offered for the best paper founded on original work, and at Niagara it was duly awarded, and then by a mere remnant withdrawn again, under circumstances that cast a deep shadow over the Association. The offer of that prize was, at Minneapolis, finally withdrawn, although there were contestants for it. It was the least that could be done, in view of the action at Niagara. There is a promise of better results in the present movement, as the expenditure of the money will be left with those who best know the demands of scientific work.

